# Intersecting social relations of care: a protocol for an ethnographic and interview study with South Asian people ageing in place with dementia

**DOI:** 10.1136/bmjopen-2024-092946

**Published:** 2024-12-09

**Authors:** Kate Gibson, Louise Robinson, Manpreet Bains, Kritika Samsi, Ana-Maria Cîrstea, Katie Brittain

**Affiliations:** 1 Population Health Sciences Institute, Newcastle University, Newcastle upon Tyne, UK; 2 Centre for Academic Primary Care, University of Nottingham, Nottingham, UK; 3 The Policy Institute, King's College London, London, UK

**Keywords:** Dementia, Aging, Caregivers, QUALITATIVE RESEARCH, Health Equity

## Abstract

**Introduction:**

People living at home with dementia are often cared for by family members, especially those from minority ethnic groups. Many people living with dementia from minority ethnic communities face barriers to accessing formal care. However, there is a paucity of dementia research, which foregrounds diversity within minority ethnic populations. This study, conducted between July 2024 and August 2026, will explore the diverse care experiences of South Asian people living with dementia. Conducted across four sites (Newcastle, Nottingham, Birmingham and London), it aims to understand how inequalities related to ethnicity intersect with other factors (eg, gender, age and class) to shape the dementia care experiences of people living in South Asian communities and apply this learning to explore how public policy and care systems could be improved to reduce health and social inequalities.

**Methods and analysis:**

In Newcastle, ethnographic research will be undertaken with up to 20 people living with dementia (or with memory concerns) in South Asian communities for a period of 12 months. The lengthy research period will enable a deep understanding about how experiences change as dementia progresses over time. In Nottingham and Birmingham, semi-structured interviews and/or focus groups will be conducted with up to 30 people living with dementia (or with memory concerns) in South Asian communities. This will enhance the data generated via the ethnographic research. Analysis will follow the principles of reflexive thematic analysis and will involve identifying themes and synthesising and theorising the data. Following this, findings will be reflected upon in 4–6 task groups convened in London, Nottingham and Newcastle with practitioners from health and social care, voluntary organisations or faith groups involved in dementia care. Task groups will focus on developing practical goals based on the research findings.

**Ethics and dissemination:**

Ethical approval for this study has been granted by Newcastle University Faculty of Medical Sciences Ethics Committee (Reference: 2773/43721). Findings will be disseminated to academics, practitioners, policymakers and members of the public via a range of channels including conferences, peer-reviewed publications, lay reports, leaflets and non-written formats such as animated videos.

**STRENGTHS AND LIMITATIONS OF THIS STUDY**

- The study’s focus on the ways in which inequalities intersect to shape individuals’ lived experience of dementia care is underexplored.
- The study’s use of ethnographic and qualitative methods across multiple sites will enhance the validity and the overall interpretation of the study.
- The emphasis on embedding in communities and cultivating trusted relationships over time, together with the flexibility offered by ethnographic methods, is particularly suited for this cohort.
- The study’s inclusion of task groups will ensure that the research findings can be actioned into relevant and practical community and policy goals.
- As this study is qualitative, our total sample of up to 50 participants will not be representative, nor will it be used to make generalisations regarding South Asian people’s experience of dementia care.

## Background

 Dementia is a national and global priority; the number of people living with dementia in the UK is projected to double in the next 30 years, with associated economic costs predicted to treble to over £50 billion.^1^ A particular research priority is inequalities in access to dementia health and social care.^2 3^ These inequalities are currently poorly described, especially (intersecting) structural inequalities relating to race and ethnicity. Historically, minority ethnic groups have been underserved in dementia research,^4^ which has disproportionally featured white, middle-class participants.^5^ Moreover, there is a lack of dementia research, which acknowledges the diverse composition of minority ethnic populations. This study will focus on people living with dementia in South Asian communities, the largest, non-white minority ethnic group in the UK.^6^ Within this cohort, there are significant inequalities; for instance, Pakistani and Bangladeshi people are more than twice as likely than Indian people to live in low-income households.^7^

Because rates of dementia among minority ethnic groups are increasing at a faster rate than the whole UK population,^8^ research involving this population is a growing subgenre of dementia research^9 10^; some of this research focuses on care. Qualitative research exploring the care experiences of Black Caribbean, South Asian and White British carers reports that varying cultural attitudes towards caregiving roles has implications for how best to support carers of people with dementia.^11^ The evidence base pertaining to this is nuanced and primarily focuses on the experiences of carers. Findings from a study exploring dementia care practices of Turkish, Pakistani and Arabic-speaking families in Denmark suggest that formal care services may not always be a culturally acceptable solution for minority ethnic families.^12^ Baghirathan *et al* find that African Caribbean, South Asian and Chinese caregivers are more likely to turn to black and minority ethnic-led voluntary organisations or religious institutions for support.^13^ Of the limited research including people with dementia, Motta-Ochoa *et al*’s ethnography, within a voluntary sector organisation supporting a ‘culturally diverse’ group of people with dementia, found that while most people living with dementia were cared for by family members, families’ use of external services helped protect them from experiencing their role as a burden.^14^ A significant issue, however, is that much ethnicity-focused research racialises, ethnicises and homogenises whole minority populations and fails to account for the ways in which ethnicity is dynamic and intersected by other factors.^10 15 16^ As Roche *et al* suggest, aggregating people with differing cultural backgrounds conceals heterogeneity.^15^ The small body of literature which specifically focuses on the experiences of South Asian people likewise identifies a lack of culturally acceptable formal care as a barrier for people living at home with dementia.^17^ Notably, South Asian carers are more likely to experience depression and stigma than their white counterparts, and within this grouping, Bangladeshi carers are three times more likely to be carers than are White British people,^18^ with Bangladeshi women in particular facing significant barriers to accessing formal support.^19^ Further research is required to explore how experiences of care are intersected by ethnicity and other social dynamics within this patterning of caring responsibilities, especially from the perspective of people living with dementia.

Aside from a limited few,^15 19 20^ there is a lack of research which scrutinises the structural relations responsible for race and ethnicity-related health disparities. Instead, research often focuses on minority ethnic groups’ lack of knowledge of dementia^21^ and lack of service engagement,^12 22^ implicitly positioning non-white cultural inadequacies as the problem.^10 23^ For example, Hossain identifies ‘limited knowledge and understanding of dementia among the South Asians’ as barriers.^21^ But without considering the extent to which *all* knowledge is culturally constructed or linking such findings to the institutional and structural inequalities that beget these issues, the implication is that minority ‘cultures’ are to blame for disadvantage and in turn become targets for intervention.^20^ As a result, the upstream conditions responsible for inequalities in dementia care, such as under-resourced health and social care systems, institutional racism and poverty, are left unchallenged.

This study aims to redress this imbalance by foregrounding diversity and heterogeneity to explore how structural factors shape care experiences among South Asian people living with dementia at home. Using the lens of intersectionality, we will scrutinise how other social dynamics (class and gender, for example) intersect with ethnicity and race to act as possible mechanisms for (dis)advantage, a hitherto underexplored area of dementia research. Based on the assumption that people are differentially located within intersecting systems of power, intersectionality considers how mutually constitutive forms of social oppression interact—or coexist—to influence experiences.^24^ Identifying the intersecting structural factors shaping the dementia care experiences of South Asian communities will add nuance to evidence informing the delivery of care to an increasingly diverse population ageing in place with dementia. To this end, our findings will help ensure health and social care provision is equitable and based on a clear understanding of the diverse populations it targets.

Our study focuses on the care experiences of South Asian people in Newcastle, Nottingham and Birmingham where Asian populations comprise the largest minority ethnic grouping (11.4%, 14.9% and 31%, respectively), the majority of whom identify as South Asian.^25^

The overarching aims of this study are to:

1. Foreground the diverse voices of South Asian people ageing in place with dementia to explore and understand how inequalities related to ethnicity and race intersect with other social dynamics to shape care experiences.
2. Apply this learning to explore how public policy and care systems could be adapted or improved to reduce health and social inequalities.

In light of these aims, the research will address the following questions:

1. What networks of care and support do South Asian people living with dementia in Newcastle, Nottingham, and Birmingham draw on to live independently at home?
2. What are the facilitators and barriers to accessing health and social care support when ageing with dementia at home for this cohort?
3. How do social structures intersect with race and ethnicity to shape the experience of dementia care among South Asian communities?

## Methods and analysis

Conducted via three work packages (WPs) across four sites (Newcastle, Nottingham, Birmingham and London), this study will use ethnographic and qualitative methods to provide an in-depth and ‘thick description’ of the contextual factors shaping South Asian peoples’ lived experience of dementia care. Key to ethnographic research, ‘thick description’ refers to collecting and interpreting data in a holistic way by considering the context of the social phenomena at hand.^26^ Conducted between July 2024 and August 2026, the comprehensive design of this study, follows a multimethod approach to data collection.^27^ This will allow people’s experiences to be examined from different perspectives, in turn providing multiple lenses through which to scrutinise the issues faced by minority ethnic populations (and the diversities between and within them).

While our study focuses on the dementia care experiences people residing in South Asian communities in Nottingham, Birmingham and Newcastle, it is not geographically bounded in the sense that it focuses explicitly on regional or place-specific aspects of dementia care. Although there are inevitable geographic specificities, structural inequalities are prevalent across multiple contexts,^28 29^ and conducting research in three sites will further insights into how such structural inequalities play out and the extent to which they are prevalent (or not) regardless of geographic location.

### Work package 1: an ethnography

#### Design

Our decision to foreground an ethnographic methodological approach is based on the premise that ethnography is a useful tool to enhance research inclusivity,^30^ especially for under-served communities.^14^ Ethnographic research relies on embedding in communities over time to cultivate trusting relationships with key participants and people connected to them. Observations, encounters and informal conversations allow for an interpretivist understanding of participants’ experiences and the co-creation of multiple, sometimes conflicting, knowledges about social phenomena.^31^

Ethnography is increasingly used in research to explore experiences of ageing,^32 33^ not least because it enables spontaneous observations and informal conversations rather than solely relying on verbal expression and memory recall, a requirement which can exclude people living with dementia.^14^ Within dementia studies, several ethnographies have been conducted in hospitals or long-term care settings as way to access context.^3034^,^38^ Although ethnographies exploring dementia in community settings are less common, DeForge *et al* effectively employ ethnographic methods in Canada to explore the contextual complexities within which familial and formal home-based dementia care is enacted (and evaluated).^39^ Their sample, however, was entirely white. Most relevant is Brijnath’s ethnography, which accounts for social and cultural complexities shaping care for people with dementia in India^40^ and the afore-mentioned ethnographic research conducted by Motta-Ochoa *et al*,^14^ who not only emphasise the usefulness of ethnography for reducing barriers to participation but also note the importance of building trust and working closely with community-based partners.

#### Sampling

A sample of up to 20 community-dwelling South Asian participants living with dementia (or have a likely diagnosis) and/or a trusted member of their support network (hereafter referred to as ‘carer’) will participate in ethnographic research. Given that an estimated one-third of the UK population with signs of dementia are living without a formal diagnosis,^41^ and the particularly low rates of dementia diagnosis within the South Asian population,^42^ we are including participants with a likely diagnosis of dementia. In terms of defining ‘likely’, we will be guided by carers, who as research suggests, are often attuned to declining memory long before official diagnosis is made.^43^ The sample will be one of convenience but will aim for diversity vis-à-vis ethnicity (eg, Indian, Bangladeshi and Pakistani), gender, household composition, educational and occupational background, and country of birth. Convenience sampling is an established strategy for recruiting excluded groups.^44^,^46^

We will build on connections established by KG who has previously conducted ethnographic research with marginalised and minoritised groups in West Newcastle, where in some wards, over one-third of the population are from black and minority ethnic backgrounds.^47^ These contacts, who are respected and well-connected across Newcastle’s South Asian community, will advocate for the research and support the research team to deliver study presentations and distribute multilingual recruitment posters and flyers in local shops, community centres and religious sites. Working with embedded informants in this way has been shown to be effective for enhancing trust and improving researcher sensitivity to the diversities of communities.^48^

#### Data collection

Initial informal interviews with participants and/or their carers will be undertaken to explore perceptions of living arrangements, care needs, future planning and participants’ social context. Thereafter, to explore participants’ everyday experiences of care, their engagement with care services (or not) and care practices within their families and social networks (and how these change over time), participants’ ‘dementia care journeys’ will be followed for 12 months. During this time, methods will be continually revised to adapt to participants’ shifting memory and capacity for engagement. Depending on their preferences, opportunities for participant observation will be pursued (eg, conducted during home visits or accompaniments to activities). Participant observation is an established method of exploring practices and experiences, which are difficult to put into words, and when conducted via informal visits will likely produce valuable verbal data too. Adapted diary-interviews^49^ and/or go-along interviews^50^ will be conducted with participants less willing to participate in participant observation. Ethnographic methods are flexible and co-produced, meaning they can be adapted to mitigate practical issues around participation and account for participant preferences. On conclusion of fieldwork, exit interviews will be undertaken with participants, informed by themes identified during the fieldwork. Detailed fieldnotes will be recorded following each research interaction. Participants will receive £20 shopping vouchers at several points in the ethnography to thank them for their time.

### Work package 2: qualitative interviews/focus groups in Nottingham and Birmingham

#### Design

To enrich data generated in WP1, enhance validity and deepen our understanding of participants’ experiences of care, additional qualitative interviews and/or focus groups will be conducted with up to 30 South Asian participants living with dementia and/or their carers in Nottingham and Birmingham.

#### Sampling

Eligible participants will be community-dwelling South Asian people living with dementia (or have a likely diagnosis) and/or their carers. Participants will be purposively sampled based on principles of maximum variation and theoretical data saturation to ensure both breadth and depth in terms of data and sample.^51^ Sampling criteria will help ensure diversity vis-à-vis representing different South Asian groups, gender, household composition, educational and occupational background, and country of birth. Furthermore, findings from WP1 will be consulted to refine the sampling approach for WP2 (ie, certain sampling criteria may be required to investigate findings from WP1 further). This will enable us to work towards ensuring we attain inductive thematic saturation.^52^ MB’s established connections with a range of South Asian community groups will be drawn on to facilitate the recruitment of participants.

#### Data collection

We will conduct semi-structured interviews or focus groups, which will enable preplanned topics to be covered, while affording ample opportunity to explore additional points brought up by participants in more depth. Topic guides will be based on initial interviews from WP1 and findings identified. Examples of key areas for discussion include everyday experiences of care and accessing formal care. This will allow us to unpick topics further and consider the transferability of findings (from WP1) to a different geographical context. This in turn will deepen insight into the complexity of social phenomena shaping participants’ experience of dementia care and strengthen the claims we can make from this study. Participants will be offered £20 vouchers for their contribution.

### Translation (WP1 and WP2)

For participants who do not speak English fluently, depending on their preference, we will work alongside paid translators, family members or trusted community partners.

### Analysis (WP1 and WP2)

Audio recordings will be transcribed verbatim. Transcripts will be checked for accuracy and anonymised directly following transcription. Anonymised fieldnotes will be typed out immediately following each research interaction.

Analysis will be undertaken throughout data collection and be documented in reflexive field diaries and post-interview summaries capturing emerging empirical patterns and themes. On completion of fieldwork, both datasets will be analysed separately following the principles of reflexive thematic analysis.^53^ Initial readings will facilitate familiarisation and lead to the generation of initial codes. Further reading and immersion will focus on developing, reviewing and refining these codes into more substantive themes and subthemes. NVivo 12 will be used to assist with this process. This iterative process of coding, reflection and theme development will be discussed within the research team throughout, ensuring the rigour and validity of data interpretation.

As this research is multimethod (characterised by the coexistence of complementary methodologies, in our case, ethnographic and interview-focused), the final stage of analysis will involve synthesising the datasets to narratively integrate the findings.^27^ Using a complementarity approach, we will focus on the relationships between our findings and identify how and why they coexist and the extent to which the findings complement, enrich and add to each other.^54^ During this process, the whole research team will meet regularly to discuss and compare themes identified in both datasets, refining and documenting themes, which cut across both WPs.^55^ Our focus will be on ‘meshing’ and ‘linking’ the data to explore how social contexts and processes ‘weave together’^56^ to shape participants’ dementia care experiences. This will allow us to capitalise on the multiple perspectives provided, reveal the multifaceted nature of social phenomena shaping dementia care experiences and thereby enhance the overall interpretation of the study.

### Work package 3: task groups (London, Nottingham and Newcastle)

Task groups will be convened with a range of stakeholders to reflect on WP1 and WP2 findings. Task Groups are facilitated focus groups where participants are presented with evidence and asked to comment on and make interpretations of evidence.^57^ Participants will include practitioners from health and social care, voluntary organisations or community and faith groups involved in dementia care. Recruitment of these participants will use KS’s extensive contacts in the dementia care workforce and contacts acquired throughout WP1 and WP2.

To offer regional variation for people across the country, we will convene approximately 4–6 Task Groups, half of which will be face-to-face in London, Nottingham, and Newcastle. We will offer a virtual option for those wishing to participate but unable to travel. We will present participants with findings from WP1 and WP2 and ask them specifically to reflect on how findings can be applied in their local context and practice. We will ask them to develop 2–3 practical and actionable goals focusing on either health, social care, community or policy. Detailed notes from the Task Groups will be taken, with permission, and participant names will be anonymised. We will develop a grid or matrix of responses to generate an overall picture, taking care to keep responses anonymous. This will facilitate understanding the contextual impact of the evidence generated from WPs 1 and 2 without breaking confidentiality.

### Patient and public involvement

The design of this study has been developed following 12 months of scoping and engagement activities conducted by KG aimed to establish collaborative relationships with community groups and people living with dementia in North East England. These activities have led to the formation of a patient and public involvement (PPI) group who have reviewed our research aims, our proposed fieldwork and knowledge exchange strategies and are providing practical guidance, for instance, relating to researcher positionality. This group is comprised of representatives from the voluntary sector and members of the local South Asian community who are well-embedded in relevant organisations and activities across Newcastle. In Nottingham and Birmingham, individuals associated with the aforementioned community groups supporting with recruitment will also adopt a PPI role. In both sites, these groups will continue to shape the proposed research as it unfolds.

Our PPI strategy is strengthened by implementing stakeholder Task Groups in WP3. The insights of this cohort will allow us to identify and translate our findings into meaningful and practical health, social care, community or policy goals.

### Dissemination

Insight gained from Task Groups will result in the delivery of relevant findings for practitioners and policymakers who are involved in the design and delivery of care for people living with dementia, especially minority ethnic communities, in a format that is most accessible to different groups.

Findings will be delivered via accessible and inclusive research outputs for members of the public. As well as lay reports, leaflets and press releases, these will include non-written formats such as delivering talks to community and religious organisations and an animated video effectively communicating our findings. Findings will also be disseminated via international and national conferences and published in peer-reviewed journals.

### Ethics

Ethical approval has been granted by Newcastle University Faculty of Medical Sciences Ethics Committee (Reference: 2773/43721) to cover all research sites.

Only participants who provide full informed consent will be recruited to take part in this study. All participants will undergo a comprehensive consent process at recruitment, which applies Mental Capacity Act (2005) key principles for the assessment of understanding, retaining and weighing up information and the communication of decisions. Due to the progressive nature of dementia, during the ethnography participants may lose capacity to consent or this capacity may fluctuate. As such, consent will be considered an ongoing process. At first research interaction, participants will be asked to confirm their wishes if they lose capacity to consent. Should a participant indicate they wish to remain in the study, the researcher will seek consent by proxy.

All participants will be fully briefed about the aims, methods and scope of the study in advance. An information sheet will clearly explain what participation entails, participants’ right to withdraw, how data will be stored, used and disseminated, and how anonymity and confidentiality will be ensured. At first research interaction, the researcher will verbally explain written documents (information sheet and informed consent form) and confirm understanding throughout. Participants who do not speak English fluently will be offered written material in their first language, or for language with no written form, in the closest alternative. These will be verbally explained by a professional interpreter or bilingual trusted contact. For the ethnography, following written consent provided in the first interview, verbal consent will be confirmed ahead of every research encounter.

## Discussion

In the UK, nearly 1 million people are estimated to be living with dementia.^1^ However, there are significant underlying health and social inequalities shaping the patterning of dementia^58^ and rates of dementia among minority ethnic groups are increasing at a significantly faster rate than the whole UK population.^8^ The ways in which race and ethnicity intersect with other inequalities to shape individuals’ lived experience of dementia care is hitherto underexplored. Minority ethnic groups are consistently excluded from research,^4^ and much ethnicity-focused research homogenises whole minority groups.^15^ Our study will focus on the diverse care experiences of people living with dementia in South Asian communities in Newcastle, Nottingham and Birmingham. Using the lens of intersectionality, we will use qualitative and ethnographic methods to explore the ways in which the care experiences of South Asian people living at home with dementia are intersected by factors such as class, gender and age. As this study is qualitative, our total sample of up to 50 participants will not be representative, nor will it be used to make generalisations regarding South Asian people’s experience of care. The aim of this study is to look towards the (intersecting) social structures, which both shape and manifest in experiences of dementia care and to provide contextual and situated knowledge, which captures the role of social structures in shaping that diversity.

Enabling independent living at home through the provision of ‘personalised’ and ‘meaningful’ care for people with dementia is a key focus of national and international policy.^59^ However, despite policy aspirations, the social care system has been described as under-resourced, unfair and unnecessarily complex.^60 61^ In the UK, the estimated 80% of people living at home with dementia are commonly cared for by (unpaid) family members, who themselves often experience economic hardship and poorer health.^62 63^ Access to formal care and support is unequally patterned, disproportionately impacting those in marginalised and minoritised social positions.^64^,^66^ To this end, our findings will add nuance to the evidence base informing the delivery of care to the rapidly ageing population living at home with dementia. Our insights about how dementia care is done and accessed in South Asian communities will impact public, policy, practitioner and academic understandings about inequalities and dementia care. This, in turn, will help ensure that opportunities to improve dementia care systems and reduce inequalities are implemented in policy and practice.
