# Supplementary material for: Intersecting social relations of care: a protocol for an ethnographic and interview study with South Asian people ageing in place with dementia
Source: BMJ Open. 2024 Dec 9;14(12):e092946. doi: 10.1136/bmjopen-2024-092946 (PMC11628994; doi:10.1136/bmjopen-2024-092946)
Supplement: Supplementary file 1 [file bmjopen-2024-092946.reviewer_comments.pdf]

PEER REVIEW HISTORY

BMJ Open publishes all reviews undertaken for accepted manuscripts. Reviewers are asked to complete a checklist review form and are provided with free text boxes to elaborate on their assessment. These free text comments are reproduced below.

ARTICLE DETAILS

Title (Provisional)

Intersecting social relations of care: a protocol for an ethnographic and interview study with South Asian people ageing in place with dementia

Authors

Gibson, Kate; Robinson, Louise; Bains, Manpreet; Samsi, Kritika; Cirstea, Ana-Maria; Brittain, Katie

---

VERSION 1 - REVIEW

Reviewer 1

Name Wilkinson, Iain

Affiliation Surrey and Sussex Healthcare NHS Trust, Medicine for Elderly

Date 23-Sep-2024

COI

I think a comment on 'likely dementia' should.be clarified. When someone has a formal diagnosis sometimes that opens up access to services. So the non formal dx is an important part of your work looking at barriers, but I just think it needs some clarity as to how you might assess likely.

Comment on numbers, presumably until data reaches a kind of saturation. Do you expect to recruit 100s to each part or 10s? I think that would be helpful to understand more.

VERSION 1 - AUTHOR RESPONSE

Reviewer Report:

1. I think a comment on 'likely dementia' should be clarified. When someone has a formal diagnosis sometimes that opens up access to services. So the non formal dx is an important part of your work looking at barriers, but I just think it needs some clarity as to how you might assess likely.

Thank you for the opportunity to clarify our understanding of 'likely dementia'. We have added the following text on p6:

Given that an estimated one third of the UK population with signs of dementia are living without a formal diagnosis (41), and the particularly low rates of dementia diagnosis within the South Asian population (42), we are including participants with a likely diagnosis of dementia. In terms of defining 'likely', we will be guided by carers, who as research suggests, are often attuned to declining memory long before official diagnosis is made (43).

2. Comment on numbers, presumably until data reaches a kind of saturation. Do you expect to recruit 100s to each part or 10s? I think that would be helpful to understand more.

For a qualitative study, our sample of up to 50 participants across WP1 and WP2 is large. We are not looking to make generalisations from this research, nor do we wish to suggest that our sample is representative of people's experiences of dementia care within South Asian communities. Our decision to recruit up to 20 participants in WP1 is a pragmatic one, shaped by a realistic estimation of gaining longitudinal access to potential participants and an anticipation that such methods will capture the rich, complex, and multi-faceted social relations shaping dementia care for South Asian people. Our decision to conduct additional qualitative interviews and/or focus groups with up to 30 participants in WP2 is intended to enhance the validity of the study and elicit a deeper, comprehensive understanding of participants' experiences of care. We would like to draw the reviewer's attention to the following text on p7:

> To enrich data generated in WP1, enhance validity, and deepen our understanding of participants' experiences of care, additional qualitative interviews and/or focus groups will be conducted with up to 30 South Asian participants living with dementia and/or their carers in Nottingham and Birmingham.
>
> ### Sampling
>
> Eligible participants will be community-dwelling South Asian people living with dementia (or have a likely diagnosis) and/or their carers. Participants will be purposively sampled based on principles of maximum variation and theoretical data saturation to ensure both breadth and depth in terms of data and sample (50). Sampling criteria will help ensure diversity vis-à-vis representing different South Asian groups, gender, household composition, educational and occupational background, and country of birth. Furthermore, findings from WP1 will be consulted to refine the sampling approach for WP2 (i.e., certain sampling criteria may be required to investigate findings from WP1 further). This will enable us to work towards ensuring we attain inductive thematic saturation (51).
